# 表观遗传机制在非小细胞肺癌靶向治疗获得性耐药中的研究进展

**DOI:** 10.3779/j.issn.1009-3419.2021.102.34

**Published:** 2021-10-20

**Authors:** 忻 艾, 燕 王

**Affiliations:** 100021 北京，国家癌症中心/国家肿瘤临床医学研究中心/中国医学科学院北京协和医学院肿瘤医院内科 Department of Medical Oncology, National Cancer Center/National Clinical Research Center for Cancer/Cancer Hospital, Chinese Academy of Medical Sciences and Peking Union Medical College, Beijing 100021, China

**Keywords:** 肺肿瘤, 靶向治疗, 耐药机制, 表观遗传, Lung neoplasms, Targeted therapy, Resistance mechanism, Epigenomics

## Abstract

驱动基因阳性的非小细胞肺癌患者能从靶向治疗中获益，但最终都不可避免地出现获得性耐药。表观遗传修饰包括DNA甲基化、组蛋白修饰、非编码RNA调控、染色质重塑等，是非小细胞肺癌靶向治疗获得性耐药的重要机制。近年来，已有研究发现表观遗传修饰的改变可以有效逆转耐药性，靶向治疗联合表观调控可能成为有前景的治疗策略。本文就非小细胞肺癌靶向治疗获得性耐药后表观遗传机制的进展进行了综述，希望为筛选优势人群、克服靶向耐药提供参考和思路。

## Abstract (en)

Patients with oncogenic driver alterations of non-small cell lung cancer (NSCLC) can benefit from targeted therapy, but acquired resistance is inevitable ultimately. Epigenetic modifications, including DNA methylation, histone modifications, non-coding RNA-mediated regulate and chromatin remodeling, are important mechanisms of acquired resistance in targeted therapy of NSCLC. In recent years, studies have found that epigenetic modifications can effectively reverse drug resistance. Targeted therapy combined with epigenetic modifications may become a promising therapeutic strategy. Here, we review the progress of epigenetic mechanism in acquired resistance of targeted therapy in NSCLC, hoping to provide ideas for screening dominant population and overcoming resistance.

肺癌是世界范围内癌症相关的主要死亡原因^[1]^。在中国，肺癌不仅是发病率最高的癌种，而且是死亡率最高的癌种^[2]^。由于临床症状的隐匿性，大多数肺癌在被发现时已处于晚期^[3]^。随着基因检测的普及和靶向药物的研发，合并致癌驱动基因的晚期非小细胞肺癌（non-small cell lung cancer, NSCLC）患者可应用靶向治疗，并显著延长了生存时间^[4]^，这些驱动基因包括表皮生长因子受体（epidermal growth factor receptor, *EGFR*）基因突变、间变性淋巴瘤激酶（anaplastic lymphoma kinase, *ALK*）基因重排、*c-ros*原癌基因1受体酪氨酸激酶（*c-ros* oncogene 1 receptor tyrosine kinase, *ROS1*）基因融合、人表皮生长因子受体2（human epidermal growth factor receptor-2, *HER2*）基因扩增和突变、间质-上皮细胞转化因子（mesenchymal-epithelial transition factor, *MET*）基因突变、扩增和重排等。然而，靶向药物后的获得性耐药限制了其疗效及应用^[5]^。

表观遗传修饰在肺癌的发生发展中发挥了重要作用。常见的表观调控包括DNA甲基化、组蛋白修饰、非编码RNA调控和染色质重塑等，可以在不改变DNA序列的情况下调控基因表达、维持基因组稳定性^[6]^。有研究^[7]^表明，表观遗传改变是NSCLC靶向治疗获得性耐药的重要机制之一，由于表观遗传改变具有可逆性，针对表观遗传的药物（表 1）可以在一定程度上延缓或逆转靶向治疗相关的获得性耐药。本文将针对表观遗传机制在NSCLC靶向治疗获得性耐药中的研究情况加以综述，以期为临床应用提供参考。

**1:** 表观遗传相关抗肿瘤药物（数据来自ClinicalTrials.gov收集截止至2021年8月22日） Epigenetic drugs against cancer (data collected by ClinicalTrials.gov as of August 22, 2021)

Target	Regulation	Drugs	NCT number (cancer types)
DNA methylation			
DNMT	Writer	Azacitidine	02009436 (NSCLC), 01928576 (NSCLC), 01926587 (MDS, AML, CML), etc.
		Decitabine	03233724 (NSCLC, EC, MPM), 02996474 (AML), 00744757 (MDS), etc.
		Guadecitabine	03913455 (SCLC), 01696032 (OC), 02348489 (AML), etc.
		NTX-301	04167917 (AML, MDS, CML), 04851834 (OC, BC).
Histone modification-acetylation			
BET	Reader	Molibresib	01943851 (MM, AML, NHL, MDS), 01587703 (NMC), 02964507 (BRC), etc.
		Birabresib	02259114 (Solid tumors), 02698189 (AML), 01713582 (AML, ALL, MM, NHL), etc.
		AZD5153	03205176 (Solid tumors), 03527147 (NHL).
		BMS-986158	03936465 (Pediatric cancer), 04817007 (MF), 02419417 (Advanced cancer), etc.
		CC-90010	04324840 (GBM), 03220347 (Solid tumors, NHL), etc.
		CPI-0610	04603495 (MF), 02157636 (MM), 01949883 (Lymphoma), etc.
		FT-1101	02543879 (AML, MDS, NHL).
		INCB057643	04279847 (MF), 02959437 (Solid tumors).
		ODM-207	03035591 (Solid tumors).
		PLX51107	04022785 (AML, MDS), 04910152 (GVHD).
		RO6870810	02308761 (AML, MDS), 03068351 (MM), 01987362 (Solid tumors), etc.
		SYHA1801	04309968 (Solid tumors).
		ZEN-3694	02711956 (PC), 03901469 (BRC), 04840589 (Solid tumors), etc.
HDAC	Eraser	Abexinostat	00724984 (Lymphoma), 03592472 (RCC), 01543763 (Solid tumors), etc.
		Belinostat	01310244 (NSCLC), 00926640 (SCLC), 00413075 (Solid tumor, lymphoma), etc.
		Bisthianostat	03618602 (MM).
		Chidamide	01836679 (NSCLC), 04582955 (BRC), 02697552 (NHL), etc.
		Citarinostat	02886065 (MM).
		Domatinostat	04874831 (MCC), 04871594 (UC), 04133948 (Melanoma), etc.
		Entinostat	00602030 (NSCLC), 02833155 (BRC), 03179930 (Lymphoma), etc.
		Givinostat	00792467 (HL), 01901432 (PV), 00606307 (PV, ET, MF), etc.
		Mocetinostat	03220477 (NSCLC), 00359086 (Lymphoma), 02236195 (UC), etc.
		Nanatinostat	05011058 (EBV+lymphoma).
		Panobinostat	04326764 (AML, MDS), 00738751 (NSCLC, HNC), 01242774 (AML), etc.
		Pracinostat	03151304 (MDS), 01912274 (AML), 01112384 (Sarcoma), etc.
		Quisinostat	02728492 (NSCLC, OC), 02948075 (OC), 00677105 (Solid tumors, lymphoma), etc.
		Resminostat	01037478 (HL), 02400788 (HCC), 01277406 (CRC), etc.
		Ricolinostat	02632071 (BRC), 02091063 (Lymphoma), 02189343 (MM), etc.
		Romidepsin	01302808 (NSCLC), 00086827 (SCLC), 01822886 (PTCL), etc.
		Tacedinaline	00005093 (NSCLC), 00005624 (MM), 00004861 (Pancreatic cancer).
		Tefinostat	00820508 (Hematological disease, Lymphoid malignancies), 02759601 (HCC).
		Tinostamustine	03903458 (Melanoma), 02576496 (Hematological malignancies), 03345485 (Solid tumors), etc.
		Trichostatin A	03838926 (Hematologic malignancies), etc.
		Valproic acid	00084981 (NSCLC), 02124174 (AML, MDS), 02068586 (Melanoma), etc.
		Vorinostat	02151721 (NSCLC), 03263936 (AML), 03742245 (BRC), etc.
		AR-42	02569320 (MM), 02282917 (VS, meningiomas), 01798901 (AML), etc.
		CXD101	01977638 (Solid tumors, lymphoma, MM).
		HG146	03710915 (MM), 04977167 (Solid tumors, lymphoma).
		KA2507	03008018 (Solid tumors).
		OKI-179	03931681 (Solid tumors).
Histone modification-methylation			
EZH	Writer	Lirametostat	02395601 (BCL), 03525795 (Solid tumors), 03480646 (PC).
		Tazemetostat	02860286 (MPM), 04624113 (HNC), 04846478 (PC), etc.
		Valemetostat	04703192 (PTCL), 04842877 (BCL), 04388852 (PC, RCC, UC).
		CPI-0209	04104776 (Solid tumors, lymphoma).
		HH2853	04390737 (Solid tumors, NHL).
		MAK683	02900651 (DLBCL).
		PF-06821497	03460977 (SCLC, PC, FL, DLBCL).
		SHR2554	04407741 (Solid tumors, lymphoma), 03603951 (Mature lymphoid neoplasms), 04355858 (BRC).
LSD1	Eraser	Seclidemstat	03600649 (Ewing or Ewing-related Sarcomas), 04734990 (CML, MDS), 03895684 (Solid tumors).
		Tranylcypromine	02261779 (AML), 02717884 (AML, MDS), etc.
		CC-90011	03850067 (SCLC), 04628988 (PC), 02875223 (Solid tumors, NHL), etc.
		IMG-7289	04262141 (ET, PV), 02842827 (AML, MDS), etc.
		INCB059872	02712905 (Solid tumors and Hematologic malignancies).
DNMT: DNA methyltransferase; BET: bromodomain and extra-terminal domain; HDAC: histone deacetylase; EZH: enhancer of zeste homolog; LSD1: lysine-specific histone demethylase 1A; NSCLC: non-small cell lung cancer; MDS: myelodysplastic syndrome; AML: acute myeloid leukemia; CML: chronic myeloid leukemia; EC: esophageal carcinoma; MPM: malignant pleural mesothelioma; SCLC: small cell lung cancer; OC: ovarian cancer; BC: bladder cancer; MM: multiple myeloma; NHL: non-hodgkin lymphoma; NMC: NUT midline carcinoma; BRC: breast cancer; ALL: acute lymphocytic leukemia; MF: myelofibrosis; GBM: glioblastoma; GVHD: graft versus host disease; PC: prostate cancer; RCC: renal cell carcinoma; MCC: merkel cell carcinoma; HL: Hodgkin lymphoma; PV: polycythemia vera; ET: essential thrombocythemia; UC: urothelial carcinoma; EBV: Epstein-Barr virus; HNC: head and neck cancer; HCC: hepatocellular carcinoma; CRC: colorectal carcinoma; PTCL: peripheral T-cell lymphoma; BCL: B-cell lymphoma; VS: vestibular schwannoma; DLBCL: diffuse large B-cell lymphoma; FL: follicular lymphoma.			

## 1 DNA甲基化与靶向药物获得性耐药

DNA甲基化是指在DNA甲基转移酶（DNA methyltransferases, DNMTs）的催化作用下，将S腺苷甲硫氨酸（S-adenosylmethionine, SAM）提供的甲基转移到DNA链上胞嘧啶第5位碳原子上，将胞嘧啶修饰为5甲基胞嘧啶（5mC）。5mC存在于CpG二联核苷，其常成簇串联排列，被称为CpG岛。启动子区CpG岛的甲基化程度可直接影响下游基因的表达，一般来讲，高度甲基化的基因处于失活状态，而低甲基化意味着基因转录^[8]^。癌细胞通常表现出以下甲基化特征：启动子的DNA高甲基化及全基因组的DNA低甲基化。全基因组的DNA低甲基化会导致基因组不稳定和致癌基因的异常激活，而启动子DNA的高甲基化可以抑制某些基因的表达，尤其是抑癌基因^[9]^。肿瘤发生过程中，启动子高甲基化可能由不同的机制引起，例如甲基胞嘧啶双加氧酶（ten-eleven translocations, TETs）家族的功能丧失或DNMTs家族的过表达。为了降低甲基化程度，许多DNA甲基转移酶抑制剂（DNA methyltransferase inhibitors, DNMTIs）被研发，包括阿扎胞苷（Azacitidine）、地西他滨（Decitabine）、Guadecitabine、Zebularine等（表 1）^[10]^。

NSCLC对靶向治疗耐药的过程中，存在DNA甲基化稳态的破坏、甲基化相关特殊耐药突变的产生和抑癌基因的失活，因此DNMTIs可通过阻断甲基化在克服耐药上发挥调节作用。

DNA甲基化稳态的破坏与NSCLC靶向治疗获得性耐药具有相关性。Terai等^[11]^将吉非替尼耐药细胞系与非耐药细胞系对比，发现640个基因的甲基化程度上调，其中29个基因出现转录mRNA的下调。研究者关注了与成纤维生长因子2-成纤维生长因子受体1（fibroblast growth factor 2-fibroblast growth factor receptor 1, FGF2-FGFR1）通路相关的两个基因：*KL*和*S100P*，使用*DNMTI*去甲基化后，*KL*和*S100P*重新表达；使用siRNA敲低*KL*和*S100P*后，细胞对吉非替尼产生耐药性。

DNA甲基化与靶向治疗获得性耐药突变*EGFR* T790M的产生具有相关性。*EGFR* T790M突变是使用一代或二代EGFR酪氨酸激酶抑制剂（EGFR tyrosine kinase inhibitor, EGFR-TKI）后常见获得性耐药的原因之一，即c.2369位置的胞嘧啶脱氨转变为胸腺嘧啶，导致790位氨基酸由甲硫氨酸代替苏氨酸。然而，上述脱氨过程后会转变为胸腺嘧啶还是尿嘧啶，取决于胞嘧啶的甲基化状态，若为甲基化的胞嘧啶，则脱氨后形成胸腺嘧啶^[12]^。研究^[12]^证实，在PC9等多个肺癌细胞系中，c.2369位置的胞嘧啶是甲基化的，EGFR-TKI（包括吉非替尼、厄洛替尼、阿法替尼、奥希替尼）的使用激活了核因子κB（nuclear factor kappa-B, NF-κB）通路，从而激活了胞苷脱氨酶（activation-induced cytidine deaminase, AICDA）的表达，AICDA导致c.2369位置的5-甲基胞嘧啶脱氨形成胸腺嘧啶，从而产生T790M突变，说明DNA甲基化与T790M突变的产生相关。

DNA甲基化与抑癌基因失活所致靶向治疗获得性耐药具有相关性（图 1）。第10号染色体缺失的磷酸酶和张力蛋白同源物（phosphatase and tensin homolog deleted on chromosome 10, *PTEN*）基因是一种抑癌基因，在许多肿瘤细胞中呈抑制状态。研究^[13]^表明，在吉非替尼耐药的细胞系中，PTEN启动子处可观测到CpG岛的甲基化状态及PTEN的低表达，用DNMTI处理耐药细胞系后，PTEN的表达恢复，且耐药细胞系对吉非替尼和厄洛替尼的敏感性也得到了恢复。死亡相关蛋白激酶（death associated protein kinase, DAPK）与细胞分化、凋亡等相关，Yang等^[14]^发现，在肺腺癌PC9细胞系中DAPK启动子呈现非甲基化状态，而在PC9/GR（存在T790M突变的吉非替尼耐药细胞系）中DAPK启动子呈现甲基化状态，经DNMTI去甲基化处理后呈现部分甲基化状态。随着甲基化状态“低-高-低”的改变，DAPK蛋白质的表达及细胞对吉非替尼的敏感性均呈现“高-低-高”趋势，提示DNMTI可以通过*DAPK*基因启动子的去甲基化作用逆转吉非替尼耐药性。综上，DNA甲基化是NSCLC靶向药物获得性耐药的机制之一，针对DNA甲基化的表观遗传调控可能是逆转耐药的有效措施。

**1:**
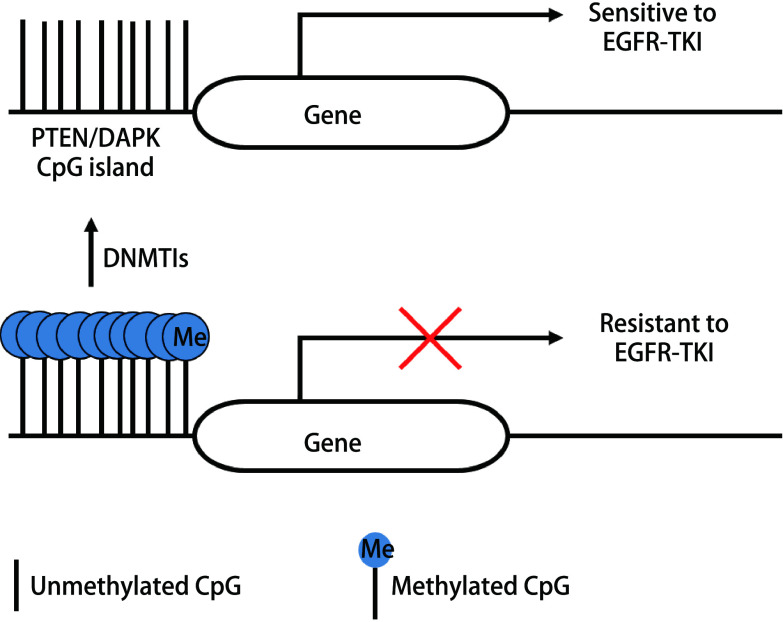
PTEN、DAPK启动子CpG岛高甲基化与EGFR-TKI耐药。TKI耐药细胞系中可观测到PTEN、DAPK启动子CpG岛高甲基化及相应抑癌基因低表达，而应用DNMTIs后可降低甲基化程度、恢复抑癌基因表达，从而克服肿瘤细胞对靶向药物的耐药性。 CpG hypermethylation of PTEN, DAPK promoter and EGFR-TKI resistance. In TKI-resistant cell lines, CpG hypermethylation of PTEN, DAPK promoter and low expression of these tumor suppressor genes can be observed. The application of DNMTIs can reduce the degree of methylation and restore gene expression, thereby overcoming drug resistance. PTEN: phosphatase and tensin homolog deleted on chromosome 10; DAPK: death associated protein kinase; EGFR-TKI: epidermal growth factor receptor tyrosine kinase inhibitor; DNMTIs: DNA methyltransferase inhibitors.

## 2 组蛋白修饰与靶向药物获得性耐药

核小体是染色质的主要结构单位，是由H2A、H2B、H3、H4四对组蛋白组成的八聚体，其外缠绕双螺旋DNA。组蛋白伸出核小体外的氨基末端可以发生翻译后修饰，包括乙酰化、甲基化、磷酸化、泛素化等，这些修饰可以改变核小体的松紧结构，从而调控基因表达^[15]^。多种蛋白酶可催化翻译后修饰，“写入”“读取”或“擦除”这些表观遗传标记，因此成为潜在的治疗靶点^[16]^。

### 2.1 组蛋白乙酰化

组蛋白乙酰化发生在赖氨酸残基上，主要与转录激活相关，乙酰化和去乙酰化可分别导致原癌基因的表达和抑癌基因的沉默，促进肿瘤的发生，由乙酰化相关的“写入”蛋白、“读取”蛋白或“擦除”蛋白协同调控^[16]^。

#### 2.1.1 乙酰化的“写入”

组蛋白乙酰转移酶（lysine acetyltransferases, KATs/histone acetyltransferases, HATs）可催化组蛋白乙酰化，其主要分为5个亚家族，分别为：HAT1（或称为KAT1）家族、Gcn5/PCAF（或称为KAT2a/KAT2B）家族、MYST（或称为KAT5）家族、p300/CBP（或称为KAT3B/KAT3A）家族、Rtt109（或称为KAT11）家族^[17]^。p300/CBP包括p300和CREB结合蛋白（CREB-binding protein, CBP），其过表达可通过组蛋白乙酰化促进肿瘤发生发展，提示不良预后。研究^[18]^发现，p300/CBP的抑制剂A485与肿瘤坏死因子相关凋亡诱导配体（TNF-related apoptosis-inducing ligand, TRAIL）联合治疗EGFR-TKI耐药NSCLC，既可短期内诱导细胞死亡，又可长期维持生长抑制作用。

#### 2.1.2 乙酰化的“读取”

含溴区结构域蛋白（bromodomain proteins, BRDs）家族的亚类溴结构域和超末端结构域（bromodomain and extra-terminal domain, BET）蛋白家族可“读取”组蛋白乙酰化标记，通过识别、结合表观遗传修饰来调节基因表达。Meng等^[19]^对比奥希替尼耐药细胞系H1975-OR与敏感细胞系H1975-P中BET的表达，发现耐药细胞系中BET的表达上调，敲除BET可显著抑制耐药细胞生长。另外，在两个细胞系中对比应用BET抑制剂JQ1，其对耐药细胞系的生长抑制作用更强。

#### 2.1.3 乙酰化的“擦除”

组蛋白去乙酰化酶（histone deacetylases, HDACs）可去除组蛋白的乙酰化标志，其主要分为5个亚家族，包括I类（HDAC1、HDAC2、HDAC3、HDAC8）、IIa类（HDAC4、HDAC5、HDAC7、HDAC9）、IIb类（HDAC6、HDAC10）、III类（SIRT家族）、IV类（HDAC11）^[20]^。由于HDACs可促进肿瘤细胞存活、增殖、转移、血管生成等，科学家研发了多种组蛋白去乙酰化酶抑制剂（histone deacetylase inhibitor, HDACi），包括伏立诺他（Vorinostat）、帕比司他（Panobinostat）、罗米地辛（Romidepsin）、恩替诺特（Entinostat）、西达本胺（Chidamide）等（表 1）^[7, 10]^。

组蛋白去乙酰化是第一代EGFR-TKI（包括吉非替尼、厄洛替尼、埃克替尼）获得性耐药的机制之一。研究^[21]^发现，将厄洛替尼与新型HDACi YF454A联合应用于耐药细胞系可导致细胞周期停滞、不可逆生长抑制及凋亡，其部分机制在于下调了*HER2*、*MET*、*AXL*、*IGF1R*等多种与耐药相关的基因表达。伏立诺他联合吉非替尼可通过诱导热休克蛋白90（heat shock protein 90, HSP90）裂解并降低其下游分子通路中与增殖相关的表达产物（包括EGFR、MET、AKT），促进细胞凋亡^[22]^。西达本胺联合埃克替尼可通过抑制RAS/MAPK、PI3K/AKT、JAK/STAT通路介导细胞周期阻滞，通过激活Caspase 3和PARP促进细胞凋亡，来提高埃克替尼对于耐药细胞系的敏感性^[23]^。肿瘤干细胞与耐药及复发相关，Bora-Singhal等^[24]^发现，HDAC11抑制剂可通过降低胚胎干细胞转录因子SOX2的表达来影响肿瘤干细胞的自我更新，恢复EGFR-TKI的敏感性。一项I期临床研究（NCT00738751）^[25]^表明，在经治的晚期NSCLC及头颈肿瘤患者中应用帕比司他联合厄洛替尼，8例*EGFR*突变肺腺癌患者的中位无进展生存期（progression-free survival, PFS）为4.7个月，中位总生存期（overall survival, OS）为41个月，其中共有4例患者曾应用过厄洛替尼。一项I期/II期临床试验（NCT01027676）^[26]^在经治的晚期NSCLC患者中应用伏立诺他联合吉非替尼，虽然研究未纳入曾应用EGFR-TKI的患者，但上述联合用药在存在*EGFR*敏感突变患者的后线治疗中，疾病缓解率（response rate, RR）可达77%，中位PFS可达9.1个月，中位OS可达24.1个月。

以奥希替尼为代表的第三代EGFR-TKI可以克服*EGFR* T790M突变，但根据AURA研究^[27]^，*EGFR* T790M阳性患者应用奥希替尼的中位PFS为9.6个月，多数患者还是不可避免地产生耐药，耐药机制包括C797S突变等。Zang等^[28]^发现，帕比司他联合奥希替尼可有效抑制多个奥希替尼耐药细胞系（包括C797S突变细胞系）中的细胞增殖和肿瘤生长，机制上与诱导高水平B淋巴细胞瘤-2基因-同源蛋白-11（B-cell lymphoma 2-like 11, BIM）相关。对于同时携带EGFR L858R/T790M/C797S突变的肺腺癌细胞系，联合应用布加替尼与伏立诺他可增强抗肿瘤作用^[29]^。

BIM属于BCL-2家族，是细胞凋亡的关键调节剂，BIM缺失可削弱凋亡能力、产生获得性耐药^[30, 31]^。上述Zang等^[28]^的研究中，敲除BIM后，帕比司他联合奥希替尼对耐药细胞的凋亡作用明显减弱。一代EGFR-TKI与HDACi联用也可通过上调BIM克服耐药，例如，伏立诺他与吉非替尼联用可显著增加BIM表达，恢复耐药细胞对吉非替尼的敏感性^[32]^。Resminostat联合吉非替尼可增加BIM水平，诱导肿瘤细胞凋亡^[33]^。伏立诺他联合二甲双胍可进一步提高BIM的表达水平，协同诱导细胞凋亡，增强吉非替尼的敏感性^[34]^。伏立诺他联合吉非替尼治疗先前接受过EGFR-TKI和化学治疗的BIM缺失的晚期NSCLC的I期临床试验（NCT02151721）^[35]^结果显示，6周治疗后疾病控制率（disease control rate, DCR）为83.3%（10/12），中位PFS为5.2个月（95%CI: 1.4-15.7），中位OS为22.1个月（95%CI: 13.5-），且不良反应耐受良好。此外，奥希替尼联合Navitoclax（BCL-2家族蛋白抑制剂）的I期临床试验（NCT02520778）正在启动。因此，HDAC及BIM均为克服EGFR-TKI获得性耐药的潜在靶点。

上皮间质转化（epithelial-mesenchymal transition, EMT）是指上皮细胞转化为间质细胞的现象，与EGFR-TKI耐药息息相关^[36]^。Weng等^[37]^的研究发现，对吉非替尼或奥希替尼耐药的NSCLC细胞存在E-cadherin水平降低并呈现EMT。HMG-CoA还原酶（HMG-CoA reductase, HMGR）抑制剂（即他汀类药物）被广泛用于治疗高脂血症，既往研究^[38]^表明HMGR抑制剂可抑制肿瘤细胞中HDAC活性，促进组蛋白乙酰化并激活抑癌基因*p21*的表达。因此，Weng等^[37]^设计了JMF3086——HDAC和HMGR的双重抑制剂，验证其可以通过表观遗传机制阻碍E-cadherin降解并恢复吉非替尼敏感性。Witta等^[39]^的II期随机对照临床研究（NCT00602030）表明，厄洛替尼联合恩替诺特对比厄洛替尼单药治疗化疗后进展的132例IIIb期/IV期NSCLC，虽然总体的中位PFS相似（分别为1.97个月和1.88个月），但对于具有高水平E-cadherin的患者来说，联合组的中位OS显著长于单药组（分别为9.4个月和5.4个月，95%CI：0.13-0.92）。

综上，组蛋白乙酰化与靶向药物获得性耐药具有相关性，多种与组蛋白乙酰化相关的治疗用药可能具有临床应用前景。

### 2.2 组蛋白甲基化

组蛋白的甲基化调控是复杂多样的，不同甲基化程度（单、双、三甲基化）和不同甲基化位点可介导基因激活或基因沉默^[6, 7, 40]^。

#### 2.2.1 甲基化的“写入”

组蛋白甲基化常发生于赖氨酸和精氨酸上，因此催化组蛋白甲基化的组蛋白甲基转移酶（histone methyltransferases, HMTs）可分为两个家族：组蛋白赖氨酸甲基转移酶（histone lysine methyltransferases, HKMTs）和组蛋白精氨酸甲基转移酶（histone arginine methyltransferases, HRMTs），其中HKMTs可分为两类：含SET结构的赖氨酸甲基转移酶（包括KMT2/MLL家族、KMT1C/EHMT2/G9a等）和唯一一个不含SET结构的赖氨酸甲基转移酶DOT1L^[40]^。

*Zeste*基因增强子同源物2（enhancer of zeste homolog 2, EZH2）是HKMTs SET结构域的组成部分，可通过组蛋白H3K27双甲基化或三甲基化介导基因沉默，EZH2表达水平的下降与肿瘤进展、治疗耐药及不良预后相关^[41]^。研究^[41]^发现，无论在细胞系还是人肿瘤标本中，EZH2的低表达均与MET介导的吉非替尼获得性耐药相关，其机制在于，MET激活下游PI3K/AKT通路，AKT可使EZH2丝氨酸磷酸化，导致MET启动子上EZH2及H3K27三甲基化水平下调，激活MET通路，从而形成恶性循环。在小鼠实验中，应用吉非替尼联合PI3K/AKT抑制剂GDC0941可增加EZH2表达，逆转EGFR-TKI耐药。EZH2抑制剂和吉非替尼联合应用还可以改善EGFR-TKI原发耐药，为治疗提供新的可能^[42]^。

EHMT2（也称KMT1C/G9a）是一种HKMTs，可通过H3K9单甲基化或双甲基化促进肿瘤侵袭和转移^[40]^。Wang等^[43]^在EGFR-TKI耐药细胞系中发现EHMT2的高表达，应用EHMT2抑制剂UNC0638可通过表观调控上调PTEN表达，显著抑制癌细胞生长。在小鼠异种移植耐药模型中，联合应用厄洛替尼和EMHT2抑制剂UNC0642可显著增加抗肿瘤作用。Chang等^[44]^发现，信号传导与转录激活因子3（signal transducer and activator of transcription 3, STAT3）可通过激活G9a，使miR-145-5p水平下降，从而促进表皮生长因子受体3（epidermal growth factor receptor 3, HER3, ERBB3）过表达，介导EGFR-TKI耐药。

#### 2.2.2 甲基化的“擦除”

催化组蛋白去甲基化的酶包括组蛋白赖氨酸去甲基化酶（histone lysine demethylases, KDMs）和组蛋白精氨酸去甲基化酶（histone arginine demethylases, RDMs），其中KDMs可分为赖氨酸特异性去甲基化酶（LSDs/KDM1家族）和含有Jumonji（JmjC）结构域的去甲基化酶（JmjC KDMs/KDM2-7家族）^[40]^。

组蛋白赖氨酸特异性去甲基化酶（lysine-specific histone demethylase, LSDs/KDM1）家族介导组蛋白去甲基化，在NSCLC中常呈过表达状态，促进肿瘤侵袭和转移^[7]^。研究^[45]^发现，长期低氧可促进NSCLC细胞对吉非替尼产生耐药，机制上与LSD1/KDM1A和PLU1/KDM5B这两种KDMs相关，LSD1抑制剂SP2509和PLU1抑制剂PBIT可逆转上述低氧介导的TKI耐药。因此，多个组蛋白甲基化调节酶可能成为克服耐药、延长生存的有效靶点。

## 3 非编码RNA调控与靶向药物获得性耐药

非编码RNA（non-coding RNAs）包括lncRNAs、microRNAs等，虽不能被转录为蛋白，但对于基因调控具有多种生物学功能，并与NSCLC耐药密切相关^[7]^。

MicroRNAs为18个-25个核苷酸组成的单链非编码RNA，可作为基因转录后调节因子，介导靶向药物获得性耐药^[46]^。例如，miR-21在耐药细胞系中过表达，与PI3K/AKT通路激活呈正相关，与抑癌基因*PTEN*、程序性死亡4基因（programmed cell death 4, *PDCD4*）的表达呈负相关，抑制miR-21可诱导耐药细胞凋亡、抑制肿瘤生长^[47]^。miR-483-3p的沉默可诱导肿瘤细胞发生EMT并产生对吉非替尼的耐药性，且miR-483-3p的发生是由于其自身启动子DNA的甲基化^[48]^。低水平miR-200可增强有丝分裂诱导基因6（mitogen-inducible gene 6, *MIG6*）的表达，介导对厄洛替尼的耐药性^[49]^。miR-142-5p可通过上调HOXD8的表达使肺癌细胞对吉非替尼产生耐药^[50]^。除上述miRNAs外，miR-214、miR-181a、miR-103、miR-203、miR-130a、miR-124等均与NSCLC靶向药物获得性耐药相关^[51-56]^。

LncRNAs是一种长度大于200 nt的线性RNA，同样具有基因调控作用^[57]^。LncRNA H19在吉非替尼耐药细胞中的表达升高，而且可以通过外泌体转移到非耐药细胞中引起更多细胞产生耐药^[58]^。Lnc00460可上调miR-769-5p，其可作用于EGFR，使肿瘤细胞获得耐药性^[59]^。LncRNA SNHG5下调可促进miR-377上调，进而通过抑制含半胱氨酸的天冬氨酸蛋白水解酶（caspase-1, CASP1）促进肺腺癌细胞对吉非替尼产生耐药^[60]^。LncRNA UCA1在吉非替尼耐药细胞中上调，与EZH2相互作用，EZH2结合在细胞周期素依赖性激酶抑制因子1A（cyclin-dependent kinase inhibitor, CDKN1A）的启动子上并介导H3K27三甲基化，减少CDKN1A的表达，引起耐药，敲低LncRNA UCA1可恢复肿瘤对吉非替尼的敏感性^[61]^。

## 4 总结及展望

本文从不同表观遗传机制出发，探讨了NSCLC靶向药物获得性耐药机制，为克服耐药提供新的理论及试验依据。然而目前，多数表观遗传调控应用于TKI耐药的研究仍处于临床前试验阶段或早期临床试验阶段，未来需关注更多临床研究数据。识别晚期NSCLC患者表观遗传紊乱模式，可以为精准医学创造机会。表观遗传调节药物与驱动基因相关靶向药物的联合使用，可能成为克服耐药的有效靶点，将给攻克耐药难关带来新的希望。相信在不久的将来，开发表观调控药物将具有更广阔的应用前景，惠及更多的肺癌患者。
